# Short- and Long-term Outcomes of Group B *Streptococcus* Invasive Disease in Mozambican Children: Results of a Matched Cohort and Retrospective Observational Study and Implications for Future Vaccine Introduction

**DOI:** 10.1093/cid/ciab793

**Published:** 2021-11-02

**Authors:** Justina Bramugy, Humberto Mucasse, Sergio Massora, Pio Vitorino, Céline Aerts, Inacio Mandomando, Proma Paul, Jaya Chandna, Farah Seedat, Joy E Lawn, Azucena Bardají, Quique Bassat

**Affiliations:** 1 Centro de Investigação em Saúde de Manhiça, Maputo, Mozambique; 2 ISGlobal, Hospital Clínic, Universitat de Barcelona, Barcelona, Spain; 3 Instituto Nacional de Saúde, Ministério da Saúde, Maputo, Mozambique; 4 Department of Infectious Disease Epidemiology, London School of Hygiene and Tropical Medicine, London, United Kingdom; 5 Maternal, Adolescent, Reproductive and Child Health Centre, London School of Hygiene and Tropical Medicine, London, United Kingdom; 6 Institució Catalana de Recerca i Estudis Avançats, Barcelona, Spain; 7 Pediatrics Department, Hospital Sant Joan de Déu, University of Barcelona, Barcelona, Spain; 8 Consorcio de Investigación Biomédica en Red de Epidemiología y Salud Pública, Madrid, Spain

**Keywords:** Group B streptococcus, GBS, neurodevelopment impairment, outcomes, mortality

## Abstract

**Background:**

Invasive group B *Streptococcus* disease (iGBS) in infancy, including meningitis or sepsis, carries a high risk of mortality and neurodevelopmental impairment (NDI). We present data on iGBS from 2 decades of surveillance in Manhiça, Mozambique, with a focus on NDI.

**Methods:**

Morbidity surveillance databases in a rural Mozambican district hospital were screened for iGBS cases. From February 2020 to March 2021, surviving iGBS patients (n = 39) plus age- and sex-matched children without iGBS (n = 119) were assessed for neurocognitive development, vision, and hearing. The role of GBS in stillbirths and infant deaths was investigated using minimally invasive tissue sampling (MITS).

**Results:**

Ninety iGBS cases were included, with most children being <3 months of age (85/90). The in-hospital case fatality rate was 14.4% (13/90), increasing to 17.8% (3 additional deaths) when considering mortality during the 6 months postdiagnosis. Fifty percent of the iGBS exposed infants and 10% of those unexposed showed any NDI. Surviving GBS conferred a 11-fold increased adjusted odds of moderate/severe NDI (odds ratio, 2.8 [95% confidence interval, .92–129.74]; *P* = .06) in children aged 0–5 years. For older children (6–18 years), no differences in NDI were found between exposed and unexposed. Motor domain was the most affected among young GBS survivors. Three stillbirths and 4 early neonatal deaths (of the 179 MITS performed) were attributed to iGBS.

**Conclusions:**

In absence of preventive strategies, such as intrapartum antibiotics, iGBS remains a significant cause of perinatal and infant disease and death. GBS also causes major longer-term neurodevelopmental sequelae, altogether justifying the need for maternal GBS vaccination strategies to increase perinatal and infant survival.

**Key Findings**

**1. WHAT IS KNOWN AND WHAT IS NEW?**

Whilst iGBS is known to be a major cause of child mortality in high income settings, population-based surveillance data are lacking in low-income settings, especially for NDI. We report on 2 decades of clinical and microbiological surveillance data, focusing on NDI outcomes from our site as part of a comparable multi-country study of NDI after GBS.

**2. WHAT DID WE DO AND WHAT DID WE FIND?**

GBS was a substantial cause of acute and short-term neonatal and infant mortality, and significant disability. Based on data from 39 survivors of infant iGBS and 119 age and sex matched children without iGBS, we found an 11-fold higher risk of developing any NDI among cases (50%) as compared to controls (10%).

**3. WHAT TO DO NOW IN PROGRAMMES?**

These data support the need for Individual care for at risk survivors of iGBS, in addition to support to families. Improved surveillance data and its use are essential to demonstrate the hidden short- and long-term burden of iGBS, particularly in LICs.

**4. WHAT NEXT IN RESEARCH?**

Operationalising routine surveillance for iGBS as part of population-based surveillance allows to establish the baseline burden prior to future preventive interventions but cannot be sustainable for GBS alone. Sentinel sites like Manhiça are suitable for pre- and post-vaccine roll out impact assessment. Use of MITS provides much more granular information on the role of GBS and other pathogens in childhood mortality.

The first 2 decades of the 21st century are indisputably characterized by a global health revolution that has led to massive improvements in health outcomes, particularly in terms of child survival [1]. Indeed, worldwide, a new baby’s chance of survival is now greater than at any time in our species’ history, and child mortality has become uncommon in many industrialized regions. The number of child deaths is rapidly decreasing worldwide, from >17 million annual deaths in the 1970s to around 5.2 million in 2019 [2]. Neonatal deaths, defined as those occurring within the first 28 days of life, have also decreased, but at a much slower pace than in older age groups, thus representing an increasingly important proportion of overall global child mortality (from ~40% in 1990 to 47% in 2019, for a total of 2.4 million deaths [3]). If we do not address these differences in mortality rates, it will be more than a century before a newborn from sub-Saharan Africa has the same survival probability as one born in Europe [1, 4].

A substantial proportion of the approximately 6700 newborn deaths that occur every day are a direct consequence of neonatal infections [5]. It has been estimated that every year, approximately 6.9 million new cases of suspected severe neonatal infection occur in low- and middle-income countries (LMICs), resulting in nearly 500 000 deaths globally [6]. Among these infections, group B *Streptococcus* (GBS) [7, 8] stands as a major pathogen linked to invasive GBS disease (iGBS) in young infants, causing a significant burden estimated at 392 000 cases of infant iGBS each year, and with a high associated case fatality rate (CFR), resulting in approximately 147 000 stillbirths and young-infant deaths annually [9] and substantial—yet insufficiently well characterized—disability among survivors.

Although GBS carriage rates vary substantially by region, approximately 18% of pregnant women globally carry GBS in their genitourinary tract [10], and this pathogen can easily be vertically transmitted to the newborn. In high-income countries, screening programmes that detect carriage among pregnant women in the weeks before delivery can specifically target those women who could benefit from intrapartum antibiotic prophylaxis, known to decrease the risk of newborn disease by approximately 20-fold. In the absence of such screening and treatment strategies, approximately 1%–2% of newborns born of a mother carrying GBS will develop severe and potentially life-threatening disease [11, 12].

In spite of the growing body of evidence that supports a greater incidence of iGBS in Africa, detailed data on GBS maternal carriage and associated infant disease (including longer-term outcomes, such as neurodevelopmental impairment [NDI]) remain scarce.

In Mozambique, a variety of studies conducted in the rural area of Manhiça have highlighted the importance of this pathogen, at least in this particular setting. In a study conducted in the years 2014–2015, 21.3% of women recruited during their last term of pregnancy were GBS carriers [13], with a predominance of serotypes V (32.4%), Ia (14.7%), and III (10.3%). Importantly, GBS isolates detected among pregnant women were fully susceptible to penicillin and ampicillin, and approximately 70% of the women tested had immunoglobulin G antibodies against GBS.

Regarding neonatal disease, a study compiling data from 2001 to 2015 described GBS as the leading cause of young-infant (<3 months) invasive bacterial disease (IBD), with an incidence of 2.7 per 1000 live births and an associated CFR of 12.3% [14]. Of note, while the incidence for other causes of IBD had decreased throughout the years, that of GBS had remained stable. Worryingly, the analysis of the GBS isolates causing infant disease for which molecular analyses were available showed that approximately 88% were highly related serotype III isolates with well-established mutations associated with reduced penicillin susceptibility [14].

Hereby, we present insights on iGBS from 2 decades of morbidity, mortality, and microbiological surveillance conducted in the town of Manhiça, southern Mozambique. Comprehensive estimation of the local and global GBS burden is useful to support policy decisions, implement available preventive strategies, and foster the development of strategies that can become a game-changer for such an important disease, such as, for instance, maternal GBS vaccines designed to protect infants [15, 16].

## AIM AND OBJECTIVES

This is part of the series “Group B Streptococcal Disease Worldwide.” The aim of this article is to use the data collected in Mozambique as part of the GBS LMIC long-term study to assess the short- and long-term burden and characteristics of GBS perinatal infections.

### Objectives

Our objectives are to (1) describe iGBS infant outcomes (onset of disease <12 months); (2) analyze NDI outcomes among iGBS survivors from Manhiça, southern Mozambique, compared to age- and sex-matched children without iGBS from the same setting; and (3) use our population-based surveillance data to describe iGBS disease during the past 2 decades, including cause of death information derived from minimally invasive tissue sampling (MITS) conducted in stillbirths, neonatal deaths (<28 days), and infant deaths (<12 months).

## METHODS

### Study Site and Surveillance System

**Figure 1.**
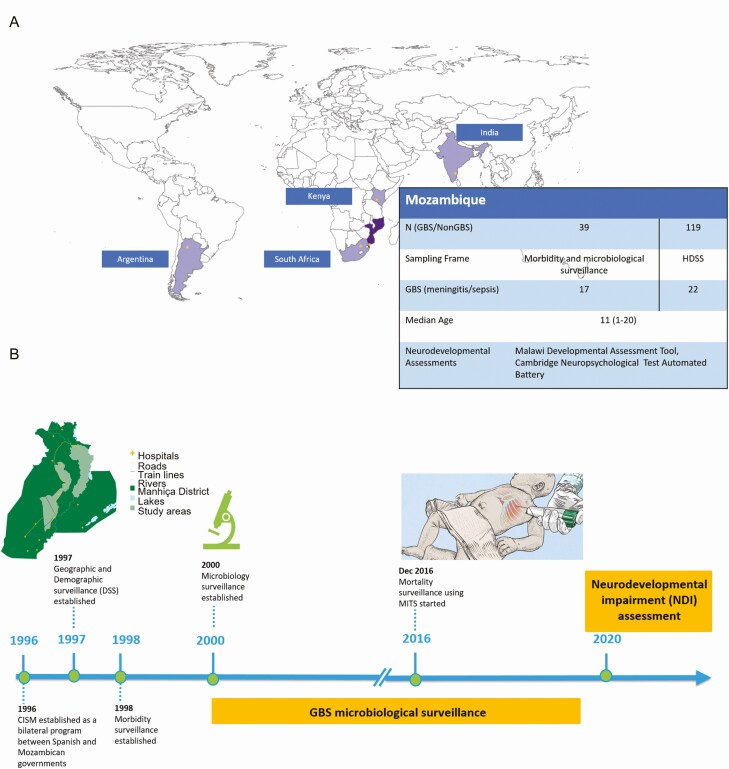
*A*, Description of Mozambique study site. *B*, Chronogram of different surveillance platforms established by Manhiça Health Research Center at the Mozambique study site, with special attention to group B *Streptococcus* surveillance. Abbreviations: CISM, Manhiça Health Research Center; DSS, Demographic Surveillance System; GBS, group B *Streptococcus*; MITS, minimally invasive tissue sampling.

This study is part of a multicountry age- and sex-matched cohort study designed to estimate the risk of long-term NDI and other outcomes in children exposed to iGBS, for which the protocol has been published previously [17]. We present data on the components conducted in Manhiça, a semi-rural town in southern Mozambique (Figure 1A). The Manhiça Health Research Center (CISM) has been running since the late 1990s as a Demographic Surveillance System (DSS) in the area and a morbidity surveillance system at the Manhiça District Hospital (MDH), across the street (Figure 1B). A detailed description of MDH, CISM, and the study area can be found elsewhere [18]. MDH is the referral hospital for the Manhiça district, covering a population of approximately 203 000 inhabitants. The MDH includes adult and pediatric wards, together with a maternity ward where between 3500 and 4000 deliveries take place annually. It also includes an outpatient department and an antenatal clinic where pregnant women are routinely followed. As part of the national policy, all pregnant women are invited to attend antenatal consultations during their pregnancy, where human immunodeficiency virus (HIV) testing and other screenings of infections and conditions are routinely offered, in addition to intermittent preventive treatment during pregnancy for malaria prevention, a disease highly endemic in the area. Manhiça district has one of the highest prevalences of HIV in the world, with HIV prevalence estimates as detected during antenatal consultations peaking at around 37% [13]. No strategy to screen for GBS carriage among pregnant women, or to prevent neonatal sepsis, is currently implemented in Mozambique.

The morbidity surveillance system has systematically collected clinical data in all children <15 years of age since its inception in 1998. A standardized admission questionnaire is completed for all pediatric admissions. Beyond this, and as part of the microbiology surveillance in place since the year 2000, a single venous blood culture is performed routinely for all admitted children <2 years of age (and for older children with hyperpyrexia or suspicion of sepsis). Upon discharge or death, a physician revises the clinical history and records the final diagnoses, using the coding system proposed by the *International Classification of Diseases, Tenth Revision*.

CISM’s databases from the DSS and the morbidity and microbiology surveillance were combined to allow a comprehensive evaluation of iGBS among sick children in the district.

### MITS for GBS-Related Mortality Surveillance

Since December 2016, Manhiça conducts MITS to eligible stillbirths and child deaths occurring within the district, as part of the Child Health and Mortality Prevention Surveillance (CHAMPS) project (https://champshealth.org/). MITS is a simplified postmortem protocolized approach to investigate cause of death, validated to substitute the complete diagnostic autopsy in all age groups (including stillbirths, neonates [19], and older children [20]). MITS includes the targeted collection of bodily fluids (blood, cerebrospinal fluid [CSF]) and tissues from key organs (liver, lungs, brain, etc) which are then subject to a thorough microbiological screening (using cultures and TaqMan Array Cards targeting >100 pathogens) and histopathological assessment. Once all results are available, a panel of local experts determines whether GBS was involved in the causal chain leading to each stillbirth or child death [21].

### Case Definitions for Included Children With iGBS and Nonexposed Children

This analysis focuses on infant iGBS (ie, <12 months of age at the moment of iGBS diagnosis), but also presents some GBS data linked to stillbirths, defined as any baby delivered with no signs of life after 28 weeks of gestation. Microbiologically confirmed iGBS was defined as a positive blood or CSF culture from any infant admitted to MDH. Early-onset disease was defined as IBD in children aged <7 days, and late-onset disease as IBD in children aged 7–89 days. We also included a group of older infants, spanning from 90 days to <12 months of age. Syndromes associated to iGBS included sepsis (if blood culture was positive) and meningitis (if CSF culture was positive), but cases with both sepsis and meningitis also occurred. Nonexposed children without GBS were recruited using the DSS databases, after randomly age- and sex-matching healthy children from the community to the identified iGBS cases. We defined as in-hospital deaths all deaths occurring during the admission and derive CFR estimates from these data. Moreover, using DSS data, we were able to evaluate postdischarge short-, mid- and longer-term mortality in iGBS survivors.

### NDI Assessments

As part of the larger study, we were able to recruit iGBS survivors and assess their neurodevelopment, with the study’s primary endpoint being any NDI. Using a matched cohort study design, we collected data on NDI. Children with no history of iGBS (henceforth, non-iGBS comparison group) were identified from the site’s DSS databases and matched to iGBS survivors (ratio 3:1) based on sex and age. Children without GBS were recruited for the study only after parents provided written informed consent.

An age-specific neurodevelopmental assessment tool, the Malawi Developmental Assessment Tool (MDAT), was used to comprehensively assess development (motor, language, social, and cognition) in children 0–5 years of age. The Cambridge Neuropsychological Test Automated Battery (CANTAB) was used to assess cognitive development in children aged 6–18 years, and an epilepsy screening questionnaire was utilized (Supplementary Table 1 summarizes all tests used in Mozambique according to age group). Anthropometric measures were also recorded during the visit. A tablet-based, custom-designed application was used to capture data through direct assessment of the child and interviews with the main caregiver, and experienced assessors, clinical psychologists, and clinicians administered the neurodevelopmental assessments. Hearing and vision were first clinically assessed; suspected impairment was referred for specialized clinical management. NDI outcomes were coded and graded by severity (mild if 1–2 standard deviations [SD] below the standardized mean, moderate if 2–3 SD, and severe if ≥3 SD) where normative populations were available, as described in the published protocol [22]. For the CANTAB, normative populations for children were only available for spatial working memory and spatial span tests [23]. Assessors were blinded to the iGBS history of the children while conducting their evaluation.

### Statistical Analysis

Statistical analyses were done with Stata version 15.2 software (StataCorp, College Station, Texas). For the retrospective analysis, study variables were counted and summarized in frequency tables. Means with corresponding SD, or medians and interquartile ranges, are presented for normally or nonnormally distributed variables, respectively. For the NDI component, baseline characteristics were compared between the iGBS survivors and non-iGBS cohort using χ ^2^ or Fisher exact test for categorical variables and difference of means for continuous variables. Linear regression was used to estimate adjusted mean differences in MDAT subscales (ages 0–5 years) and CANTAB tests (6–18 years), accounting for the matching variables age and sex. The risk of neurodevelopmental outcomes (moderate/severe, any NDI) was quantified for the iGBS survivors and non-iGBS cohorts, stratified by age bands (0–5 years, 6–18 years). Logistical regression adjusting matching variables estimated the association of NDI outcomes. A probability of < .05 was considered statistically significant.

**Ethical Considerations**

For the field data collection in LMICs, the overarching protocol for the observational study was granted ethical approval at the London School of Hygiene and Tropical Medicine (approval number 16246). The institutional review board in Mozambique reviewed and approved the protocol and data analysis (reference number 98/CNBS/2019). Data from CHAMPS were collected and shared under the Mozambican Ethics Committee’s approval (reference numbers 285/CNBS/16 and 484/CNBS/18).

## RESULTS

### GBS in Manhiça Infants, 2000–2020

During the 2 decades spanning from 1 January 2000 to 31 December 2019, 60 659 children aged <15 years were admitted to MDH, of whom 21 208 (34.9%) were infants, including 2006 (9.5%) aged <7 days; 4576 (21.6%) aged 7 to <90 days, and 14 626 (69.0%) aged 3 to <12 months. A total of 90 infants (0.42% of all infants) were confirmed as iGBS cases, with 29 cases occurring in the first 7 days of life, 56 in the period 7 to <90 days, and 5 additional cases in children aged 3 to <12 months. Of those, 65 (72.2%) were characterized as sepsis only, 8 (8.9%) as meningitis only, and 17 (18.9%) as both sepsis and meningitis. Meningitis as a syndrome was more commonly, but not exclusively, found in the age group 7 to <90 days, similar to dual infections (sepsis plus meningitis).

Of the 90 iGBS cases, there were 13 in-hospital deaths, yielding a CFR of 14.4%, significantly higher than the CFR for all other admitted infants (1054/21 118 [5.0%]; *P* < .0001). Three additional deaths were identified through the DSS among these 77 survivors of iGBS, one occurring 24 hours after hospital discharge, one 48 hours after discharge, and one 147 days after the iGBS episode. The extended CFR (including deaths occurring within 6 months postdischarge) was 17.8% (16/90). Table 1 summarizes some characteristics of the iGBS cases, according to age at detection.

**Table 1.** Characteristics of Infant Invasive Group B Streptococcus Infections, According to Age Group

Characteristic	EOD GBS (Age <7 d)	GBS LOD (Age 7 to <90 d)	Older-Infant GBS Disease (Age 3 to <12 mo)	Total iGBS Infants (Age <365 d)
Sex, female	10/29 (34.5)	29/56 (51.8)	1/5 (20.0)	40/90 (44.4)
Age, d, median (IQR)	1 (0–2)	13 (8–17)	224 (181–230)	8.5 (4–16)
Clinical characteristics				
Respiratory distress	15/28 (53.6)	17/56 (30.4)	1/5/20.0)	33/89 (37.1)
Unable to feed	12/29 (41.4)	17/56 (30.4)	1/5 (20.0)	30/90 (33.3)
Cough	3/29 (10.3)	23/56 (41.1)	5/5 (100)	31/90 (34.4)
Fever (≥37.5°C)	17/29 (58.6)	48/56 (85.7)	5/5 (100)	70/90 (77.8)
Diarrhea	1/29 (3.5)	3/56 (5.4)	2/5 (40.0)	6/90 (6.7)
Vomiting	1/29 (3.5)	5/56 (8.9)	1/5 (20.0)	7/90 (7.8)
Convulsions	4/29 (13.8)	6/55 (10.9)	0/5 (0)	10/90 (11.2)
Bulging fontanelle	1/29 (3.5)	6/56 (10.7)	0/5 (0)	7/90 7.8)
Clinical syndrome				
Only meningitis	2/29 (6.9)	6/56 (10.7)	0/5 (0)	8/90 (8.9)
Only sepsis	22/29 (75.9)	38/56 (67.9)	5/5 (100)	65/90 (72.2)
Both	5/29 (17.2)	12/56/21.4)	0/5 (0)	17/90 (18.9)
Duration of admission, days, median (IQR)	4 (2–6)	6.5 (4–9)	7 (4–9)	6 (3–8)
In-hospital mortality	7/29 (24.4)	5/56 (8.9)	3/5 (60.0)	13/90 (14.4)
Delayed mortality	0/29 (0)	2/56 (3.6)	1/5 (20.0)	3/90 (3.3)

Data are presented as no./No. (%) unless otherwise indicated.

Abbreviations: EOD, early-onset disease; GBS, group B *Streptococcus*; iGBS, invasive group B *Streptococcus* disease; IQR, interquartile range; LOD, late-onset disease.

### Neurodevelopmental Issues Detected Among GBS Survivors

**Figure 2.**
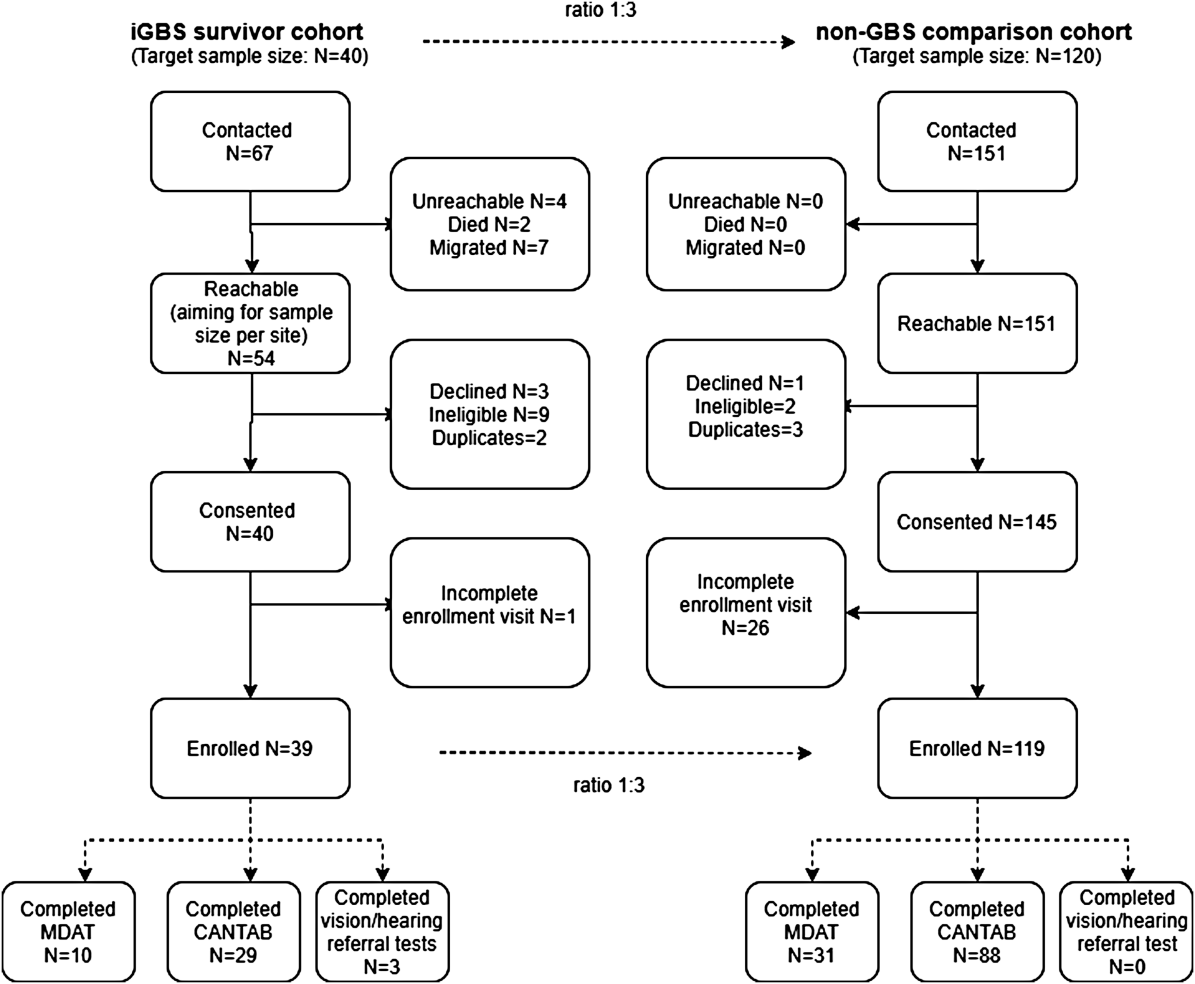
Participant flow diagram. Of 67 invasive group B *Streptococcus* survivors contacted, 39 consented for participation and completed the assessment. Of 151 matched unexposed children contacted for participation, 121 children consented and completed neurodevelopmental, vision, and hearing assessments. Abbreviations: CANTAB, Cambridge Neuropsychological Test Automated Battery; GBS, group B *Streptococcus*; iGBS, invasive group B *Streptococcus*; MDAT, Malawi Developmental Assessment Tool.

During the period February 2020 to March 2021, we attempted to contact and recruit 67 iGBS survivors and 151 age- and sex-matched children without iGBS. Thirty-nine iGBS survivors were finally recruited and followed up to assess long-term outcomes, and their results were compared to those of 119 age- and sex-matched children without iGBS (Figure 2). Baseline characteristics between the iGBS survivors and non-GBS cohorts were similar, except for birthweight (*P* < .001) and highest educational attainment for main caregiver (*P* < .001) (Supplementary Table 2). MDAT and CANTAB were administered to 10 and 29 iGBS survivors, respectively. Moderate and/or severe impairment was identified in 40% (4/10) and 4% (1/29) of children 0–5 years old and >5 years old, respectively (Table 2). After adjusting for matching variables, iGBS was associated with a 10.9 (95% confidence interval [CI], .92–129.74) increased odds of moderate and/or severe NDI compared to the comparison group, although differences did not reach statistical significance (*P* = .058) in 0- to 5-year-olds. No moderate and/or severe NDI was measured using the CANTAB, irrespective of iGBS history. Mild NDI was identified among 1 of 10 (10.0%) and 2 of 29 (8.7%) iGBS survivors aged 0–5 years and 6–18 years, respectively (Table 2). The odds of any NDI in children 0–5 years old with history of iGBS was 8.41 (95% CI, 1.47–47.98); there was no association of any NDI and iGBS history in older children (odds ratio, 0.63 [95% CI, .21–1.86]). Moderate and/or severe NDI was detected in 4 of 15 (26.7%) and 1 of 22 (4.5%) of iGBS-meningitis and iGBS-sepsis survivors, respectively. iGBS children with cognitive impairment also had motor impairment on the MDAT. There were no behavioral problems identified in either the iGBS survivors or non-GBS cohorts. Two iGBS survivors were identified with severe hearing problems and one iGBS survivor was identified with severe hearing and mild vision problems based on clinical determination.

**Table 2.** Severity of Impairment in Invasive Group B Streptococcus (GBS) Survivors Versus Age- and Sex-Matched Non-GBS Controls

Age Group and Level of Impairment	iGBS Meningitis (n = 6)	iGBS Sepsis (n = 4)	iGBS Survivors (n = 10)	Non-GBS Cohort (n = 31)
0–5 years				
Overall NDI				
Moderate/severe	3 (50.0)	1 (25.0)	4 (40.0)	1 (3.4)
Mild	1 (16.7)	0 (0.0)	1 (10.0)	2 (6.9)
Motor				
Moderate/severe	2 (28.6)	1 (25.0)	3 (27.2)	3 (9.1)
Mild	1 (14.3)	0 (0.0)	1 (9.1)	1 (3.0)
Cognition				
Moderate/severe	2 (33.3)	1 (25.0)	3 (27.2)	3 (9.1)
Mild	1 (16.7)	0 (0.0)	1 (9.1)	1 (3.0)
Any behavioral problems	0 (0.0)	0 (0.0)	0 (0.0)	0 (0.0)
Multidomain impairment	4 (66.7)	1 (25.0)	5 (50.0)	3 (9.7)
	iGBS Meningitis (n = 11)	iGBS Sepsis (n = 18)	iGBS Survivors (n = 29)	Non-GBS Cohort (n = 88)
6–18 years^a^				
Overall NDI				
Moderate/severe	1 (11.1)	0 (0.0)	1 (4.3)	5 (6.0)
Mild	1 (11.1)	1 (7.1)	2 (8.7)	14 (16.9)
Cognition				
Moderate/severe	0 (0.0)	0 (0.0)	0 (0.0)	5 (6.0)
Mild	1 (10.0)	2 (11.8)	3 (11.1)	14 (16.9)
Any behavioral problems	0 (0.0)	0 (0.0)	0 (0.0)	0 (0.0)
Multidomain impairment	1 (9.1)	0 (0.0)	1 (3.4)	5 (5.7)

Data are presented as No. (%) unless otherwise indicated.

Abbreviations: GBS, group B *Streptococcus*; iGBS, invasive group B *Streptococcus* disease; NDI, neurodevelopmental impairment.

^a^Motor impairment severity could not be estimated in 6- to 18-year-olds because standard reference populations were not available. Overall NDI was based on 2 Cambridge Neuropsychological Test Automated Battery cognitive tests, hearing, and vision.

Children aged 0–5 years with a history of iGBS passed 12 fewer items on the MDAT compared to the non-GBS cohort, although this was statistically not significant (*P* = .46) (Supplementary Table 3). Exposed children seemed to show a trend toward worse performances in the gross and fine motor and language domains compared to the unexposed children, but this did not reach statistical significance. Among the children with no impairment, the same tendency was observed. Notably, there was a significant difference between mean gross motor score between iGBS-exposed children and non-GBS children (mean difference, –5.0 [95% CI, –8.6 to –1.4]; *P* = .01) (Supplementary Table 3). There was no difference in performance in the social domain between the 2 cohorts.

iGBS children who completed the CANTAB generally performed similarly to the comparison cohort, except for one test. In the test for sustained attention (rapid visual information processing) the GBS cohort had a slower response time compared to the non-GBS group (Supplementary Table 4).

### Postmortem Surveillance of GBS as a Cause of Death

During the period 9 December 2016 through 25 May 2020, 222 MITS were conducted in stillbirths or children under the age of 5 having died in Manhiça. Eighty percent (179/222) of all MITS conducted corresponded to stillbirths and infants, including 75 stillbirths (33.8%); 61 newborns in their first day of life (27.5%), 18 newborns aged 1 to <7 days (8.1%); 6 newborns aged 7–28 days (2.7%); and 19 postneonatal infants (8.6%). Among these, GBS was detected as part of the multiple microbiology and/or immunohistochemical tests in 9 cases (4.1%), but was only deemed to play a role in the causal chain leading to death in 7 cases (3.2%) (Table 3). Cases were detected in 3 of 75 (4.0%) of the stillbirths, 3 of 61 (4.9%) of newborns in their first day of life, and 1 of 18 (5.5%) of the children 1 to <7 days old. These 7 deaths occurred at the hospital and affected children at term and with adequate birthweight, except for one case of unknown gestational age but with birthweight of 2000 g. Of note, GBS disease causing stillbirths or neonatal deaths was not related to other coinfecting pathogens, nor other clear comorbidities, and in all but one case, GBS was considered the sole cause in the chain of events leading to death. In all but one of the cases, strong evidence of chorioamnionitis or funisitis was present. For all cases, GBS was detected in the lung tissues, either by molecular methods or in the immunohistochemistry. All cases had at least 2 tissues/bodily fluids where GBS could be detected.

**Table 3.** Characteristics of Group B Streptococcus–Associated Deaths as Part of Postmortem Surveillance in Manhiça

Characteristic	Case 1	Case 2	Case 3	Case 4	Case 5	Case 6	Case 7
Sex	Female	Male	Female	Female	Male	Female	Male
Age at death	2 h	14 h	24 h	12 h	NA	NA	NA
Age group	<24 h	<24 h	1 to <7 d	<24 h	Stillbirth (fresh)	Stillbirth (fresh)	Stillbirth (macerated)
Birthweight, g	2800	3000	2700	2000	3200	3000	2800
Gestational age, wk	41	Unknown	38	37	Unknown	38	39
Mode of delivery	Vaginal	Cesarean	Vaginal	Vaginal	Vaginal	Vaginal	Vaginal
Maternal HIV status	Negative	Positive	Positive	Negative	Positive	Negative	Negative
HIV status of case	Negative	Negative	Negative	Negative	Negative	Negative	Unknown
Hours between death and MITS	29	10	3	5	7	21	11
Location of death	Hospital	Hospital	Hospital	Hospital	Hospital	Hospital	Hospital
Hours in hospitalization	2	14	23	Unknown	NA	NA	NA
Underlying cause of death (*ICD-10* code)	P23.3	P36.0	P36.0	P36.0	P23.3	P23.3	P23.3
Underlying cause of death (text)	Congenital infection	NN sepsis	NN sepsis	NN sepsis	Congenital infection	Congenital infection	Congenital infection
Immediate cause of death (*ICD-10* code)	P36.0	…	…	…	…	…	…
Immediate cause of death (text)	NN sepsis	None	None	None	None	None	None
Sites where iGBS was detected	Blood (culture); CSF (TAC), lung (TAC, IHC), brain (IHC), placenta (IHC)	Lung (TAC, IHC); umbilical cord (IHC)	Blood (TAC), lung (TAC), CSF (TAC), placenta (16S PCR)	Blood (TAC), lung (TAC; IHC),	Blood (culture), lung (TAC), CSF (TAC), placenta (IHC)	Lung (TAC), placenta (IHC), umbilical cord (IHC)	Blood (TAC), lung (TAC), placenta (IHC)
Maternal GBS suspected/confirmed disease	Chorioamnionitis	Intrapartum fever	Chorioamnionitis	None	Chorioamnionitis	Chorioamnionitis	Chorioamnionitis
Other coexisting pathogens	No	No	No	No	No	No	No
No. of steps in chain of events leading to death	2	1	1	1	1	1	1

All Child Health and Mortality Prevention Surveillance (CHAMPS) patients are de-identified and their data associated to a study number, so as to guarantee their privacy and that of their families.

Abbreviations: CSF, cerebrospinal fluid; HIV, human immunodeficiency virus; *ICD-10*, *International Classification of Diseases, Tenth Revision*; iGBS, invasive group B *Streptococcus* disease; IHC, immunohistochemistry; MITS, minimally invasive tissue sampling; NA, nonapplicable, NN, neonatal; PCR, polymerase chain reaction; TAC, TaqMan Array Card.

## Discussion

This analysis, presenting a variety of data related to iGBS disease (including long-term outcomes) and death as detected during a 20-year period in the rural district of Manhiça, southern Mozambique, confirms the high burden posed by GBS in early infancy, both as a cause of acute morbidity and mortality, but also importantly, as a cause of longer-term neurodevelopmental sequelae. Importantly, and as opposed to other pathogens typical of the neonatal period and early infancy, whose burden and impact seem to have decreased throughout the years, no major changes have occurred for iGBS, the incidence of which has remained relatively constant during the last 20 years (data not shown) [14]. Although total numbers may appear relatively low (n = 90 cases of iGBS, out of a total of 21 208 infants admitted to hospital), it is important to consider that our case definition of iGBS required the microbiological confirmation of this pathogen in either blood or CSF, and that the yield of single low-volume blood cultures may be suboptimal despite this test having been conducted systematically among all admitted infants. The overall proportion among infants (0.4%) is also falsely reassuring, given that iGBS was rare beyond the age of 3 months. Thus, our estimates are surely a severe underestimation of the true burden, but allow us to infer that this pathogen, for which no specific preventive measures are currently in place in Mozambique, plays a significant role in threatening the health of the youngest. Our postmortem data reinforce this hypothesis, given that around 1 of 20 of the stillbirths and early neonatal deaths studied could be attributed specifically and solely to this pathogen. Given that our morbidity surveillance can only document information regarding sick children attending the hospital, it is important to highlight the role of mortality surveillance through postmortem investigations, which can provide additional and actionable information on the role of GBS, including much-needed visibility on cause of death among stillbirths, an area traditionally neglected and for which data scarcity is disheartening [24].

Our data also confirm the significant long-term burden (including postdischarge mortality) of GBS disease. The specific assessment of neurodevelopmental problems following survival of iGBS, when compared to control children, confirms the highest odds of developing NDI in children 0–5 years of age [25]. Surprisingly, there was so difference in odds of impairment between the 2 groups of children aged 6–18 years. It may be that impairments that are identified at earlier ages may resolve by school age. Alternatively, the lack of standardized populations for some of the motor and cognitive tests may have limited our ability to appropriately classify impairment severity; however, the mean scores of the test were not statistically different between the 2 groups. Additionally, motor sequelae were also often associated to cognitive impairment. Given that most of the severe impairment cases detected in Manhiça correspond to children for whom GBS meningitis was detected, our findings confirm the importance of including support to and aftercare of bacterial meningitis survivors, not only to improve GBS long-term outcomes, but also as one of the pillars of the Defeating Meningitis Roadmap [26].

Our study has strengths but is also affected by a variety of limitations. Strengths include the long-term surveillance (including postmortem), population-based capture, and efforts to increase multicountry comparability of the NDI data, which is a challenge in all such studies, especially for older ages (see the articles by John et al and Harden et al in this supplement). The study includes a variety of limitations, such as, for instance, our inability to control for other potential confounders, specifically gestational age at birth. Changes in our surveillance over time with alterations in datasets affecting comparability over time may also have affected our results. Although morbidity and microbiological surveillance have occurred uninterruptedly during these 2 decades, efforts have differed, for example, in terms of conducting lumbar punctures among newborns with suspected IBD, or in terms of the contamination rates of blood cultures experienced throughout the years, which could have hampered our real capacity to detect true pathogenic microorganisms. However, despite this potential for underreporting, underdetection, or misdiagnosis, the fact that we were able to comprehensively assess 20 consecutive years of infant admissions is commendable, together with our capacity to link postdischarge outcomes using the DSS databases or tracking survivors to evaluate how GBS may have hampered their normal development. Interestingly, while the different tests utilized were able to pinpoint cases that were moderate and/or where severe neurodevelopmental issues had occurred, these same tests failed to detect milder issues. Although it is possible that the team of psychologists performing those tests may have encountered particular difficulties when delivering them, it is also conceivable that such tests may have been culturally inappropriate or underperforming in this setting. For the CANTAB, in particular, there were only norms available for 2 of the 5 cognitive tests and no motor tests, which may result in underestimating impairment. Further evaluation of these and other similar tests may help understand these puzzling results. In addition, we were only able to screen for severe hearing and visual problems and therefore unable to quantify mild-to-moderate outcomes. Although we were only able to include 39 iGBS cases out of the original 67 iGBS survivors detected, we do not feel that there were any biases between those cases included and those not included.

These data highlight the need for programmes to establish adequate follow-up of iGBS survivors, both in the short term but also the long term, in order to prevent and monitor the potential emergence of NDI. Additionally, programmes need also to support families who will have to bear the longer-term consequences of this early-life infection. From a health systems perspective, operationalizing routine surveillance for iGBS as part of population-based surveillance allows to establish the baseline burden prior to future preventive interventions but cannot be sustainable for GBS alone. Sentinel sites like Manhiça are suitable for pre- and postvaccine rollout impact assessment.

## Conclusions

Our long-standing surveillance platforms in Manhiça district have highlighted the major burden and impact of GBS as a pathogen, and the perils this particular bacterium poses for fetal and child survival. Indeed, iGBS remains an important cause of mortality and long-term sequelae among survivors. In the absence of preventive strategies based on screening and treating pregnant women, which are extremely challenging to implement in a typical rural African setting, a maternal vaccination strategy could become the game-changer that this disease requires. While we accelerate progress for vaccines, we also need to advance routine data surveillance to be able to track the impact of future vaccine introductions.

## Supplementary Data

Supplementary materials are available at *Clinical Infectious Diseases* online. Consisting of data provided by the authors to benefit the reader, the posted materials are not copyedited and are the sole responsibility of the authors, so questions or comments should be addressed to the corresponding author.

ciab793_suppl_Supplementary_Materials

## Notes

***Author contributions.*** J. E. L. conceived the idea and obtained the funding for the larger study in which this analysis is included. J. B., H. M., P. V., S. M., A. B., C. A., and Q. B. were responsible for the invasive group B *Streptococcus* (GBS) and neurodevelopmental impairment (NDI) data. Q. B. and I. M. provided Child Health and Mortality Prevention Surveillance (CHAMPS) data on stillbirths and infant deaths for Mozambique. P. P., J. C., and F. S. analyzed the NDI-related data. J. B. and Q. B. prepared the first draft of the manuscript. J. B., A. B., and Q. B. had final responsibility for the decision to submit the manuscript for publication. All authors provided input to the content of the manuscript, reviewed each draft, and reviewed and approved the final version.

***Supplement sponsorship.*** This supplement is sponsored by Bill & Melinda Gates Foundation.

***Acknowledgments.*** The authors thank the Mozambican team working in the CHAMPS project that provided data on GBS infection among stillbirths and children with a fatal outcome.

***Data sharing statement.*** The specific dataset regarding infant invasive GBS in the period 2000–2020 from Manhiça is available (de-identified) upon request. Unpublished data, including new datasets on NDI or stillbirths, from the CHAMPS project, require communication with investigators leading these specific studies.

***Financial support.*** This work was supported by the Bill & Melinda Gates Foundation to the London School of Hygiene and Tropical Medicine (LSHTM) (grant number NV-009018; principal investigator [PI]: J. E. L.). P. V., S. M., I. M., J. B., and A. B. report subawards from LSHTM to Mozambique and European and Developing Countries Clinical Trials Partnership (EDCTP)–funded fellowship (number 2836; PI: Marieke Van der Zalm). Q. B. reports subawards from LSHTM to ISGlobal and Centro de Investigação em Saúde de Manhiça (CISM)/Fundação Manhiça, Mozambique. H. M. reports subawards from LSHTM to Mozambique. CHAMPS is supported by the Bill & Melinda Gates Foundation (grant number OPP1126780). J. B. holds an EDCTP-funded fellowship (number 2836; PI: Marieke Van der Zalm). CISM is supported by the government of Mozambique and the Spanish Agency for International Development. ISGlobal receives support from the Spanish Ministry of Science and Innovation through the Centro de Excelencia Severo Ochoa 2019–2023 program (CEX2018–000806-S), and support from the Generalitat de Catalunya through the CERCA program.

***Potential conflicts of interest.*** F. S. reports being funded by the Economic and Social Research Council in collaboration with the UK National Screening Committee (NSC) to do a PhD project to explore the use of international data to inform GBS screening in the UK; reports travel support to present at a GBS meeting at Universidade Federal de São Paulo; serves as a member of steering group on a pilot study on the correlates of maternal GBS protection; and reports being employed by the UK NSC, which developed the maternal GBS screening policy recommendation in the UK, all outside the submitted work. All other authors report no potential conflicts of interest.
