# Supplementary material for: Short- and Long-term Outcomes of Group B Streptococcus Invasive Disease in Mozambican Children: Results of a Matched Cohort and Retrospective Observational Study and Implications for Future Vaccine Introduction
Source: Clin Infect Dis. 2021 Nov 2;74(Suppl 1):S14–23. doi: 10.1093/cid/ciab793 (PMC8776307; doi:10.1093/cid/ciab793)
Supplement: ciab793_suppl_Supplementary_Materials [file ciab793_suppl_supplementary_materials.docx]

**Supplement Title:** Every Country, Every woman, Every Child; Group B Streptococcal Disease Worldwide

**Title**: Mozambican infants and GBS invasive disease: long-term outcomes based on two decades of surveillance and implications for future vaccine introduction

**Short title**: iGBS disease in Mozambican infants

**Authors**: Justina Bramugy^#1^, Humberto Mucasse^#1^, Sergio Massora^1^, Pio Vitorino^1^, Céline Aerts^2^, Inacio Mandomando^1,3^, Proma Paul^4,5^, Jaya Chandna^4,5^, Farah Seedat^4,5^, Joy E Lawn^4,5,^ Azucena Bardají^¶1,2,^ Quique Bassat*^¶1,2,7,8,9^

* Corresponding author

# These authors share primary authorship (joint first authors) on this work

¶ These authors are joint senior authorship (joint senior authors) on this work.

**Affiliations:**

1. Centro de Investigação em Saúde de Manhiça (CISM), Maputo, Mozambique
2. ISGlobal, Hospital Clínic, Universitat de Barcelona, Barcelona, Spain
3. Instituto Nacional de Saúde, Ministério da Saúde, Maputo, Mozambique
4. Department of Infectious Disease Epidemiology, London School of Hygiene & Tropical Medicine, London, United Kingdom
5. Maternal, Adolescent, Reproductive & Child Health (MARCH) Centre, London School of Hygiene & Tropical Medicine, London, United Kingdom
6. Department of Medical Statistics, London School of Hygiene & Tropical Medicine, London, United Kingdom
7. ICREA, Pg. Lluís Companys 23, 08010 Barcelona, Spain
8. Pediatrics Department, Hospital Sant Joan de Déu (University of Barcelona), Barcelona, Spain
9. Consorcio de Investigación Biomédica en Red de Epidemiología y Salud Pública (CIBERESP), Madrid, Spain

Table of Contents

[Supplementary tables 3](#_Toc80865344)

[Supplementary Table 1: NDI assessments, by age and domain 3](#_Toc80865345)

[Supplementary Table 2: Description of baseline characteristics in iGBS survivors and non-iGBS group (among those with valid NDI assessments) 4](#_Toc80865346)

[Supplementary Table 3: Effect of iGBS on MDAT outcomes, Mean difference, 0-5^‡^ years old 5](#_Toc80865347)

[Supplementary Table 4: Effect of iGBS on CANTAB outcomes, 6-18 years old 6](#_Toc80865348)

[STROBE Statement—Checklist of items that should be included in reports of cohort studies 7](#_Toc80865349)

# Supplementary tables

# Supplementary Table 1: NDI assessments, by age and domain

| Motor and Cognition |  |
| --- | --- |
| ≤ 5 years old | MDAT |
| ≥ 6 years old | CANTAB |
| Hearing – all ages | Clinician screening + clinical determination |
| Vision – all ages | Clinician screening + Peek acuity app |

MDAT Malawi Developmental Assessment Tool; CANTAB Cambridge Neuropsychological Test Automated Battery

# Supplementary Table 2: Description of baseline characteristics in iGBS survivors and non-iGBS group (among those with valid NDI assessments)

|  | **iGSB survivors (N=39)** | **Non-iGBS comparison (N=119)** | **p-value** |
| --- | --- | --- | --- |
| **Clinical presentation, n (%)**  Sepsis  Meningitis | 22 (56.4)  17 (43.6) |  |  |
| **GBS age, n (%)**  Early-onset  Late-onset | 7 (17.9)  32 (82.1) |  |  |
| **Age in years, mean (SD)** | 11.1 (5.8) | 11.1 (4.9) | 0.977 |
| **Sex, n (%)**  Female  Male | 20 (51.3)  19 (48.7) | 63 (52.9)  56 (47.1) | 0.857 |
| **Gestational age, n (%)**  ≥37  Don’t know | 25 (92.6)  2 (7.4) | 104 (88.1)  14 (11.9) | 0.505 |
| **Birthweight, n (%)**  ≥2500g  <2500g  Don’t know | 24 (61.5)  5 (12.8)  10 (25.6) | 36 (30.2)  5 (4.2)  78 (65.6) | <0.001 |
| **Highest education for main caregiver, n (%)**  No formal education  Primary  Secondary  Higher education (University/technical/) | 7 (18.0)  18 (46.2)  10 (25.6)  4 (10.3) | 90 (75.6)  28 (23.5)  0 (0.0)  1 (0.84) | <0.001 |

# Supplementary Table 3: Effect of iGBS on MDAT outcomes, Mean difference, 0-5^‡^ years old

|  | non-GBS | | iGBS survivors | |  |  |
| --- | --- | --- | --- | --- | --- | --- |
|  | N | Mean (SD) | N | Mean (SD) | Difference in mean scores (95% CI) | p-value |
| Total cohort | | | | |  | |
| MDAT total score | 36 | 118.8 (40.9) | 12 | 106.5 (50.6) | -12.2 (-45.3, 20.9) | 0.46 |
| MDAT gross motor | 36 | 31.3 (12.2) | 12 | 26.9 (11.1) | -4.3 (-12.4, 3.7) | 0.28 |
| MDAT fine motor | 36 | 29.7 (14.1) | 12 | 25.2 (12.5) | -4.6 (-14.0, 4.8) | 0.33 |
| MDAT language | 36 | 29.8 (13.6) | 12 | 27.4 (11.2) | -2.3 (-11.2, 6.6) | 0.59 |
| MDAT social | 36 | 27.9 (12.1) | 12 | 12.0 (10.6) | -0.9 (-9.0, 7.1) | 0.81 |
| No impairment cohort | | | | |  | |
| MDAT total score | 28 | 139.7 (20.9) | 6 | 125.5 (31.1) | -10.5 (-23.6, 2.7) | 0.11 |
| MDAT gross motor | 28 | 36.4 (4.2) | 6 | 30.5 (7.4) | -5.0 (-8.6, -1.4) | 0.01 |
| MDAT fine motor | 28 | 35.0 (8.1) | 6 | 32.2 (9.3) | -2.1 (-7.0, 2.7) | 0.38 |
| MDAT language | 28 | 35.6 (6.1) | 6 | 31.5 (8.5) | -2.8 (-7.8, 2.3) | 0.27 |
| MDAT social | 28 | 32.6 (5.5) | 6 | 31.3 (8.1) | -0.6 (-4.7, 3.6) | 0.78 |

*adjusted for matching factors age and sex

‡includes xx 6 year olds and xx year old who completed an MDAT

# Supplementary Table 4: Effect of iGBS on CANTAB outcomes, 6-18 years old

|  | non-GBS | | iGBS survivors | |  |  |
| --- | --- | --- | --- | --- | --- | --- |
|  | N | Mean (SD) | N | Mean (SD) | Difference in mean scores (95% CI) | p-value |
| Total cohort | | | | |  | |
| Attention and Psychomotor Speed | | | | | | |
| Motor Screening Task (mean time to respond correctly) | 84 | 1133.3 (313.0) | 27 | 1067.4 (518.7) | -46.9 (-203.19, 109.36) | 0.55 |
| Rapid Visual Processing | 80 | 0.8 (0.1) | 25 | 0.8 (0.1) | -0.001 (-0.041, 0.038) | 0.95 |
| Motor response speed (Mean response to stimulus time) | 85 | 347.0 (94.2) | 27 | 318.3 (95.9) | -27.07 (-62.4, 8.24) | 0.13 |
| Memory | | | | | | |
| Paired Associates Learning Test (visual memory (mean number of attempts) | 81 | 2.0 (1.7) | 25 | 2.0 (1.9) | 0.004 (-0.73, 0.74) | 0.99 |

*adjusted for matching factors age and sex

# STROBE Statement—Checklist of items that should be included in reports of cohort studies

|  | Item No | Recommendation | Page No |
| --- | --- | --- | --- |
| **Title and abstract** | 1 | (*a*) Indicate the study’s design with a commonly used term in the title or the abstract | 1 |
|  |  | (*b*) Provide in the abstract an informative and balanced summary of what was done and what was found | 1-2 |
| Introduction | | | |
| Background/rationale | 2 | Explain the scientific background and rationale for the investigation being reported | 4 |
| Objectives | 3 | State specific objectives, including any prespecified hypotheses | 5 |
| Methods | | | |
| Study design | 4 | Present key elements of study design early in the paper | 6 |
| Setting | 5 | Describe the setting, locations, and relevant dates, including periods of recruitment, exposure, follow-up, and data collection | 6-7 |
| Participants | 6 | (*a*) Give the eligibility criteria, and the sources and methods of selection of participants. Describe methods of follow-up | 7 |
|  |  | (*b*)For matched studies, give matching criteria and number of exposed and unexposed |  |
| Variables | 7 | Clearly define all outcomes, exposures, predictors, potential confounders, and effect modifiers. Give diagnostic criteria, if applicable | 7 |
| Data sources/ measurement | 8* | For each variable of interest, give sources of data and details of methods of assessment (measurement). Describe comparability of assessment methods if there is more than one group | 7 |
| Bias | 9 | Describe any efforts to address potential sources of bias | 7 |
| Study size | 10 | Explain how the study size was arrived at | 8 |
| Quantitative variables | 11 | Explain how quantitative variables were handled in the analyses. If applicable, describe which groupings were chosen and why | 8 |
| Statistical methods | 12 | (*a*) Describe all statistical methods, including those used to control for confounding |  |
|  |  | (*b*) Describe any methods used to examine subgroups and interactions |  |
|  |  | (*c*) Explain how missing data were addressed | 8 |
|  |  | (*d*) If applicable, explain how loss to follow-up was addressed |  |
|  |  | (*e*) Describe any sensitivity analyses |  |
| Results | | |  |
| Participants | 13* | (a) Report numbers of individuals at each stage of study—eg numbers potentially eligible, examined for eligibility, confirmed eligible, included in the study, completing follow-up, and analysed |  |
|  |  | (b) Give reasons for non-participation at each stage | 9 |
|  |  | (c) Consider use of a flow diagram |  |
| Descriptive data | 14* | (a) Give characteristics of study participants (eg demographic, clinical, social) and information on exposures and potential confounders | 9 |
|  |  | (b) Indicate number of participants with missing data for each variable of interest |  |
|  |  | (c) Summarise follow-up time (eg, average and total amount) |  |
| Outcome data | 15* | Report numbers of outcome events or summary measures over time | 9 |

| Main results | 16 | (*a*) Give unadjusted estimates and, if applicable, confounder-adjusted estimates and their precision (eg, 95% confidence interval). Make clear which confounders were adjusted for and why they were included |  |
| --- | --- | --- | --- |
|  |  | (*b*) Report category boundaries when continuous variables were categorized | 9-10 |
|  |  | (*c*) If relevant, consider translating estimates of relative risk into absolute risk for a meaningful time period |  |
| Other analyses | 17 | Report other analyses done—eg analyses of subgroups and interactions, and sensitivity analyses | 9-10 |
| Discussion | | | |
| Key results | 18 | Summarise key results with reference to study objectives | 11 |
| Limitations | 19 | Discuss limitations of the study, taking into account sources of potential bias or imprecision. Discuss both direction and magnitude of any potential bias | 11/12 |
| Interpretation | 20 | Give a cautious overall interpretation of results considering objectives, limitations, multiplicity of analyses, results from similar studies, and other relevant evidence | 12 |
| Generalisability | 21 | Discuss the generalisability (external validity) of the study results | 13 |
| Other information | | | |
| Funding | 22 | Give the source of funding and the role of the funders for the present study and, if applicable, for the original study on which the present article is based | 15 |
